# Epileptic Neuronal Networks: Methods of Identification and Clinical Relevance

**DOI:** 10.3389/fneur.2013.00008

**Published:** 2013-03-01

**Authors:** Hermann Stefan, Fernando H. Lopes da Silva

**Affiliations:** ^1^Department of Neurology, University Hospital ErlangenErlangen, Bavaria, Germany; ^2^Centre of Neuroscience, Swammerdam Institute for Life Sciences, University of AmsterdamAmsterdam, Netherlands; ^3^Department of Bioengineering, Instituto Superior Técnico, Lisbon Technical UniversityLisbon, Portugal

**Keywords:** epileptic networks, neurophysiological classification, MEG/EEG, basic concepts, clinical approaches

## Abstract

The main objective of this paper is to examine evidence for the concept that epileptic activity should be envisaged in terms of functional connectivity and dynamics of neuronal networks. Basic concepts regarding structure and dynamics of neuronal networks are briefly described. Particular attention is given to approaches that are derived, or related, to the concept of causality, as formulated by Granger. Linear and non-linear methodologies aiming at characterizing the dynamics of neuronal networks applied to EEG/MEG and combined EEG/fMRI signals in epilepsy are critically reviewed. The relevance of functional dynamical analysis of neuronal networks with respect to clinical queries in focal cortical dysplasias, temporal lobe epilepsies, and “generalized” epilepsies is emphasized. In the light of the concepts of epileptic neuronal networks, and recent experimental findings, the dichotomic classification in focal and generalized epilepsy is re-evaluated. It is proposed that so-called “generalized epilepsies,” such as absence seizures, are actually fast spreading epilepsies, the onset of which can be tracked down to particular neuronal networks using appropriate network analysis. Finally new approaches to delineate epileptogenic networks are discussed.

## Epileptic Neuronal Networks: Basic Concepts, Structure, and Dynamics

Seminal descriptions of neuronal networks in which neurons are the elementary units that transmit signals through synaptic contacts were performed by Ramón y Cajal (1894). The concept of neuronal networks has occupied a prominent role in the Neurosciences since. Research into how neuronal networks are interconnected forming the wiring structure of the brain has been a constant thread along the years. An important question has been to find rules that link structural connectivity of neuronal networks with information flow and processing in such networks. A model of how information may flow in cortical networks was proposed by Abeles (1991), Abeles and Gerstein (1998) who introduced the concept of “synfire chains” meaning synchronous working chains of neurons in a network, i.e., sets of interconnected neurons that participate in common tasks. He elaborated this concept further in what he called Corticonics where insights from anatomical and physiological studies are combined with mathematical and computer modeling to obtain quantitative descriptions of cortical functions. These notions have been explored in modern neural network modeling. At the level of the organization of the whole brain the network concept has been extended, among others by Mesulam (1990), describing “local networks” (engaged in modality-specific processing such as analysis of shape, spatial location, and object identification in the visual modality) and “large-scale networks” that incorporate numerous parallel lines of communication with multiple cross-links, enabling integrative processing. The complexity of the organization of these networks of the brain has been compared with that of other large-scale networks, such as the World-Wide Web, the Internet, social networks, or metabolic networks (Jeong et al., 2000), and has been the object of similar mathematical analyses based on topological properties; graph analysis is an example of this approach. In this way the notions used in these mathematical analyses have been adopted in the description of neuronal networks of the brain, and terms as “nodes” and “hubs” have entered the field of the neurosciences. Thus concepts from graph theory are being used to represent neuronal networks: a neuron is denominated a node and a neuronal network consists of nodes connected by links or edges; highly connected nodes are called hubs and an uninterrupted sequence of links forms a path; questions such as how the flow of information takes place from a node to another, can be analyzed by finding the possible paths in a graph. The structure of these networks deviates from random; this structure has some properties of “small-world” networks where any two nodes may be connected by short paths and where a few “hubs” may dominate the whole connectivity of the network (Watts and Strogatz, 1998). In such a structure the number of links of a node may follow a power-law which characterizes the so-called “scale-free” networks which are heterogeneous. Small world networks are hypothesized to optimize rapid synchronization transfer creating a balance between local processing and global integration (Meador, 2011). We should note that in current applications of these concepts in the field of neurophysiology, the “nodes” correspond simply to the sites where signals are recorded, be it using EEG, MEG, or functional magnetic resonance imaging (fMRI), and not to the neuronal elements as such. This implies that there is an enormous distance between those “nodes” and the neuronal reality.

A particular feature of some neuronal networks is that these are interconnected by means of re-entrant connections, i.e., that some nodes tend to receive connections from other nodes to which they project by relatively short paths, some of which have been well characterized both anatomically and physiologically such as the cortico-thalamic-cortical system (Steriade, 2001), and the entorhinal – hippocampal-entorhinal system (Kloosterman et al., 2004). A few hubs may dominate the whole connectivity of the network, and if these hubs would represent neuronal features with a high degree of excitability, the latter may be rapidly distributed throughout the whole network. Furthermore if the network possesses re-entrant properties an even larger network may display this high degree of excitability with complex dynamics.

Whereas the historical approach to understand higher level operating principles in the brain was to consider it subdivided into anatomical regions with local functional properties, the current approach, inspired by the theoretical analysis of complex networks, as described above, is to emphasize networks interactions and connectivity at short and long range.

These theoretical considerations provide a convenient approach to better understand the pathophysiology of epilepsies. In this context, however, it is essential to go forward from the description of network connectivity and structural properties, as presented above, to the dynamic dimension, i.e., to the study of the activity of the networks as function of time. A fundamental characteristic of these neuronal networks is that their dynamics are essentially non-linear given the non-linear transfer properties of neuronal elements.

The dynamics of the population of neurons that constitute the neuronal networks can be considered at different spatial scales: microscopic, mesoscopic, and macroscopic. The last two levels are particularly relevant with respect to the dynamics of EEG or MEG signals in general, and in the case of epileptic activity in particular. A variety of molecular processes at the microscopic (cellular) level may lead to changes in the stability of neuronal networks causing epileptiform seizures, that become manifest at the mesoscopic and macroscopic levels. A generalized concept is that seizures occur in strongly coupled neuronal networks due to a shift in the dynamical balance between excitatory and inhibitory processes with a predominance of the former. In terms of the mathematical theory of complex non-linear systems we may state that such networks display bistability, i.e., they may feature two stable operational states that may exist simultaneously for the same set of system parameters. One of these states is the normal, inter-ictal state, and the other is the epileptic or ictal state of the network. The transition between the two states is called a dynamical bifurcation (Lopes da Silva et al., 2003). In epileptic brain certain networks have abnormal parameters at the molecular and cellular levels, due to genetic or to acquired pathogenic factors, rendering some essential parameters, that control network stability, extremely vulnerable to the influence of exogenous and endogenous factors, such that this kind of bifurcations may occur easily. In this way abnormal oscillations and other events, such as epileptiform spikes, may occur in hubs of these neuronal networks with abnormal parameters.

At the local neuronal network level, some hubs constituted by neurons and associated glia constitute oscillatory systems that became increasingly coupled at the transition to a seizure, thereby recruiting more distant neuronal networks, constituting complex oscillatory circuits, which can be recognized by EEG or MEG recordings (Zhang et al., 2011). Accordingly, circuits of this kind have been described in several forms of epilepsy, such as in the thalamocortical system involved in Absence epilepsies (Meeren et al., 2002, 2005; Suffczynski et al., 2004), and also in several other forms of epilepsy as discussed by Halasz (2010) for rolandic epilepsy (inner part of sylvian fissure), Landau Kleffner syndrome (perisylvian opercular structure and/or posterior part of first temporal convolution), electrical status epilepticus in sleep (perisylvian area, bilateral widespread involvement of cortical mantle, thalamic mediodorsal nucleus), Lennox–Gastaut syndrome (diffuse bi-synchronous epileptogenic system and cortical excitation with augmented cortico-thalamic oscillations), nocturnal frontal lobe epilepsy (frontal medial and orbital surfaces). Also McIntyre and Gilby (2008) described in various models of temporal lobe epilepsy the recruitment of the parahippocampal cortices including piriform, perirhinal, and entorhinal cortex in addition to the hippocampus proper. Along the same line of thought Spencer (2002) put forward the concept of human epilepsy as a disorder of large neural networks, and Avanzini et al. (2012) proposed the term “system epilepsies” to describe some types of epilepsy that depend on the dysfunction of specific functional neural systems. Clinical and network analytical studies are required to advance detection of such dysfunctional specific systems, and characterize more precisely their abnormal structure and dynamics.

## Epileptic Neuronal Networks: Causality, Linear and Non-Linear Methods, and New Approaches

**Figure 1:**
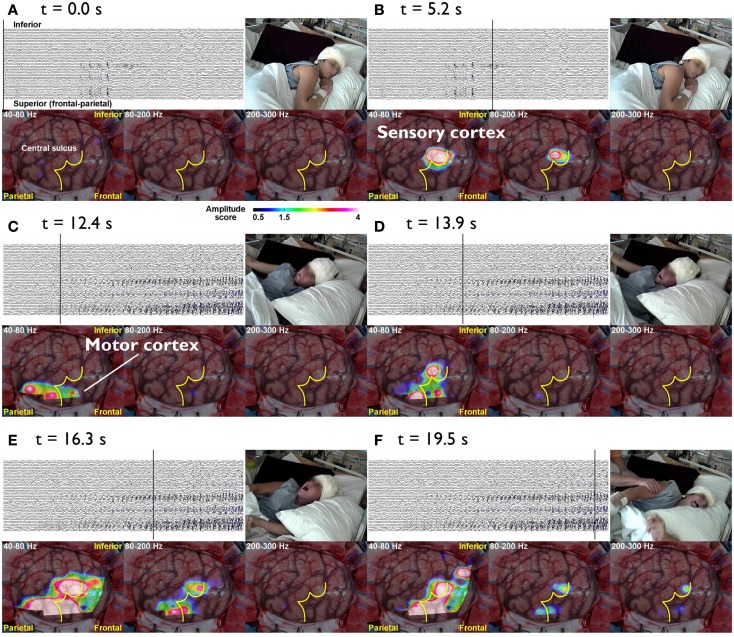
**The cursor on the EEG indicates the time of the video and topographies**. The color bar at the bottom indicates the amplitude score, where scores ≥1.5 are considered to be a significant increase from the inter-ictal period. **(A)** *t* = 0.0 s The patient lies still on the bed without symptoms. **(B)** *t* = 5.3 s During the diffuse EEG attenuation period, the patient complains of abnormal sensation in the left hand. There is an increase in the amplitude in 80–200 and 200–300 Hz bands over the sensory cortex of the left hand. **(C)** *t* = 15.5 s After 40–80 and 80–200 Hz activities start building up in the EEG, they gradually spread anteriorly toward the motor cortex of the left hand and tonic flexion of the left arm is seen. **(D)** *t* = 23.6 s Subsequently, the activities within all three bands (40–300 Hz) gradually increase in amplitude and spread to adjacent areas. Activities at 40–80 and 80–200 Hz also spread to the inferior rolandic region. However, even when the seizure becomes secondarily generalized, the HFOs are confined to the rolandic region (**E,F**) (Akiyama et al., 2011).

Epileptic conditions have to be characterized on the basis of clinical evidence, but a comprehensive analysis of the brain systems responsible for epileptic manifestations resorts to neuroimaging techniques that may reveal structural abnormalities, and to the EEG, MEG, and fMRI which can reveal the underlying dynamics. Here we concentrate on general aspects of methodologies aiming at characterizing epileptogenic networks. This implies *functional connectivity mapping* to determine the dynamics of epileptiform activities displayed as patterns of interactions between anatomically connected neural nodes responsible for these abnormal activities. Current methodologies allow a direct evaluation of correlations between EEG seizure activities, their propagation dynamics on the one hand, and the evolution of clinical signs on the other, observed using combined EEG-video recording. This is nicely illustrated in the case of patients with intractable Jacksonian seizures in whom intra-cranial EEG recordings (iEEG) were made in order to assess the indication of surgery (Akiyama et al., 2011). During the Jacksonian seizures High Frequency Oscillations (HFOs > 40 Hz) started in the sensory cortex and propagated to the motor cortex when ictal motor signs occurred, but as the seizure progressed ictal HFO spread or reverberated into the rolandic region; further when the seizure became secondarily generalized the ictal HFOs were limited to the Rolandic region (Figure 1).

Considering that the main objective of this paper is to examine evidence for the concept that epileptic activity should be envisaged in terms of functional connectivity and dynamics of neuronal networks, we emphasize here approaches that are derived, or related to the concept of causality, as formulated by Granger (1998) in econometrics. In short, according to Ganger causality an observed time-series *x*(*t*) can be considered the cause of another series *y*(*t*) if knowledge of the past values of *x*(*t*) improves the prediction of *y*(*t*).

The “directed transfer function” (DTF) extends Granger causality to multichannel EEG/MEG data (Kaminski and Blinowska, 1991) and has been applied to estimate functional connectivity in epilepsy (Franaszczuk and Bergey, 1998) and more recently by Dai et al. (2012) as discussed more in detail below. It should be noted, however, that DTF represents a linear combination of causal relations, not only along direct pathways, but also along indirect pathways. This led to the development of another measure, the so-called “direct DTF” (dDTF), which emphasizes direct associations over indirect ones (Korzeniewska et al., 2003). The latter, however, has the limitation that it needs relatively long signal epochs to be estimated reliably. To minimize the effects of non-stationary behavior of EEG/MEG signals, several methods have been developed, among which the method of short-time DTF (SDTF). By combining dDTF and SDTF new measures were proposed: the SdDTF which estimates direct causal influences between signals, not mediated by other signals, in short-time epochs, and the Event-related causality (ERC) which estimates event-related changes in SdDTF (Korzeniewska et al., 2008). This has been applied mainly in the analysis of task-related changes in EEG or MEG signals, particularly in the high frequency gamma range during cognitive tasks.

It should be added that the methodologies described above are based on the assumption that the relationships between EEG/MEG signals are linear; this may be an acceptable approximation in many cases, although during epileptic seizures it is doubtful whether the linear assumption always holds. Therefore non-linear methods were developed with the objective of estimating the coupling between different EEG/MEG signals in general. A first group of these methods was based on the estimation of mutual information (Mars and Lopes da Silva, 1983) and on non-linear regression (Lopes da Silva et al., 1993; Wendling et al., 2001; Kalitzin et al., 2007) applied to EEG or MEG signals. A second group of methods, was based on tools imported from the field of non-linear dynamical systems and chaos theory (Lehnertz, 1999; Iasemidis, 2003). Related to this class of methods two other may be distinguished, namely: phase synchronization (PS) methods (Bhattacharya et al., 2001; Rosenblum et al., 2004), generalized synchronization (GS) methods (Arnhold et al., 1999; Stam and van Dijk, 2002), and more recently directed phase lag index (dPLI; Stam and van Straaten, 2012). The former estimate the instantaneous phase of each signal and then compute a quantity based on co-variation of extracted phases to determine the degree of coupling between signals. GS methods also consist of two steps: the reconstruction of state space trajectories from time-series signals and the computation of a similarity index on reconstructed trajectories. The dPLI characterizes spatial and temporal patterns of phase relations in functional brain networks.

**Figure 2:**
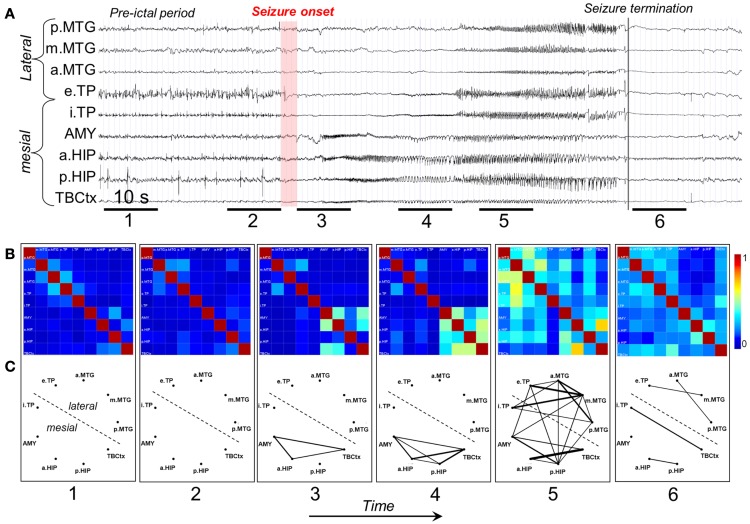
**Characterization of epileptogenic networks in the temporal lobe during the transition from pre-ictal to seizure activity**. **(A)** Intracerebral EEG recording performed in a patient with mesial TLE. **(B)** Color-coded nonlinear correlation matrices obtained from the pairwise computation of nonlinear correlation coefficient h2 over six different 10-s intervals chosen during the pre-ictal period (1, 2), the ictal period (3, 4, 5), and after seizure termination (6). **(C)** Graphical representation in which the lines indicate “abnormally strong” couplings between the two considered structures (graph nodes). Only significantly high interdependencies are represented (i.e. h2 values ≥0.32. This value corresponds to the average h2 value computed over the interictal period ±2 SD). Line thickness is proportional to h2 values.

A study of Wendling et al. (2009) is particularly interesting because these authors compared directly several methods of estimating functional connectivity between EEG/MEG signals, namely (a) linear (cross-correlation, cross-spectral analysis – coherence and phase), (b) non-linear regression (mutual information, h2 association index), (c) PS, and (d) GS, applied to a well defined data set. To make the comparison these authors built computer models of interconnected neuronal networks with defined coupling parameters, that can generate oscillatory activity typical of epileptic seizures. This model-based methodology allows establishing at will the degree of coupling between the different neuronal networks that generate the EEG signals. In this way the coupling between the signals of different networks can be estimated and the computed values obtained using different methods can be directly compared among themselves, and with the values of the coupling parameters established *a priori*. This comparison revealed that there was no “ideal” method, i.e., none of the methods performed better than all the other ones in all nine studied situations. Nevertheless, regression methods (linear or non-linear) showed sensitivity to the coupling parameter in all tested models with average or good performance, what leads to the conclusion that these are robust, and it is advisable to first apply these regression methods in order to characterize functional brain connectivity, under normal or pathologic conditions. In any case it is useful in practice to compare the results of different measures to get more reliable estimates of the coupling of interest. Figure 2 shows examples of the results of the application of non-linear regression analysis to intra-cerebral EEG signals identifying network associations around the moment of seizure onset.

In the last decade new techniques have entered the field based on the application of MRI: namely fMRI, particularly in conjunction with EEG, and diffusion-based tractography imaging (DTI). Regarding fMRI, and in the context of determining pathways of propagation of epileptic activity in neuronal networks, Dynamic Causal Modeling (DCM) applied to the interpretation of hemodynamic signals (BOLD) is being extensively used to determine the patterns of interaction between different neuronal networks (Friston et al., 2003).

In an animal experimental model of absence epilepsy, this methodology has been integrated with associated EEG signals (David et al., 2008). In this case the performances of DCM and Granger causality estimates were compared, showing approximately similar results. In human epilepsy these methodologies were applied recently in epileptic patients with Hypothalamic Hamartomas and were able to yield plausible estimates of seizure propagation pathways (Murta et al., 2012). DTI is based on the principle of anisotropic diffusion of water molecules in white matter tracts throughout brain tissue. In a study of children with temporal lobe epilepsy displaying spikes over the Rolandic region identified in the MEG, the hypothesis that the latter occurred due to activity propagating along neural aberrant pathways connecting the temporal lobe and the Rolandic cortex appeared plausible according to the DTI analysis (Bhardwaj et al., 2010).

## Epileptic Neuronal Networks: Clinical Queries and Practical Relevance

### Networks in focal cortical dysplasias and other lesion-related epilepsies

To put in evidence functional dynamics of neuronal networks engaged in epileptic seizure activity the study of Focal Cortical Dysplasias (FCD), Dysembryoplastic NeuroEpilethelial Tumors (DNET), and Periventricular Nodular Heterotopias (PNH), which frequently are associated with pharmaco-resistant epilepsy, is particularly enlightening. These lesions may be synaptically connected with other neuronal networks, such that the epileptic activity may propagate along the connecting pathways constituting an “epileptogenic network” (Aubert et al., 2009), or otherwise may stay confined to the region of, and around, the FCD lesion. Interestingly, anatomical alterations in tissue microstructure adjacent to some FCDs were detected using DTI-MR imaging. These overlapped with the localization of clusters of equivalent dipoles of epileptiform spikes (Widjaja et al., 2009).

Therefore it is most relevant to determine the functional organization of these epileptogenic networks, since this may give useful indications for a possible surgical intervention and the corresponding prognosis. Different methods have been applied to estimate the functional connectivity of neuronal networks in these cases. Using depth EEG registrations (stereoencephalography) functional analysis of multiple EEG signals was performed using non-linear regression (h2 association index) by Valton et al. (2008), and by computing the so-called “Epileptogenicity index” (Aubert et al., 2009). In the former study Valton et al. (2008) applied this methodology to analyze depth seizure EEG signals in a patient with bilateral PNH. Non-linear regression analysis revealed a large epileptogenic network extending beyond the PNH and involving remote cortical structures. The fact that this is a vast epileptogenic network may account for surgical failures in patients with this kind of heterotopias.

Aubert et al. (2009) used depth electrodes (stereoencephalography) and the “Epileptogenicity index” from Bartolomei et al. (2008). This index is based on the spectral content of high frequency components [beta: (12.4–24 Hz) + gamma: (24–90 Hz)] relative to lower frequency components. It accounts for both the propensity of a brain area to generate high frequency oscillations and the time for this area to get involved in the seizure. In this study depth EEG signals of several cortical areas were analyzed; the authors found in one group of patients (31% of the patients) one strictly localized epileptogenic zone, while a second group (61%) displayed more than one epileptogenic network. The success of surgery was related to the extent of the epileptogenic network. This implies that it is important to determine with the highest precision the extent of the epileptogenic network while planning a surgical intervention.

This study illustrates the clinical relevance of making a functional analysis of the neuronal networks associated with an epileptogenic lesion using quantitative methods of EEG signal analysis, and that the “epileptogenicity index” may be a useful tool even in cases where a relatively small number of EEG signals are available. In the case of Mesial Temporal Lobe Epilepsies (MTLE) extended networks outside the temporal lobe may be involved what led Ryvlin and Kahane (2005) to coin the denomination “Temporal-plus Epilepsy” where epileptiform activity appears in multiple brain lobes in addition to the temporal lobe.

The importance of differentiated “primary and secondary” inter-ictal spike activity for the identification of ictal and propagated epileptic activity was pointed out by Badier and Chauvel (1995).

The determination of the extent of an epileptogenic network may also be obtained from recordings of inter-ictal spikes, either using EEG or MEG, since it is known that the propagation patterns of spikes may give valuable information about neural networks associated with epilepsy (Spencer, 2002) and to the outcome of epilepsy surgery (Alarcon et al., 1997; Hufnagel et al., 2000; Schulz et al., 2000). Indeed inter-ictal EEG (reviewed in Rodin et al., 2009) and MEG spikes (Tanaka et al., 2010) may be considered biomarkers of epileptogenic networks; more recently, in the same context, a novel kind of biomarker – High Frequency Oscillations, i.e., HFOs (ripples and fast ripples), – has raised a lot of interest and its role is the object of investigation, reviewed by Jacobs et al. (2012). In order to perform this kind of analyses using scalp EEG and/or MEG recordings it is necessary to combine information about electric and/or magnetic fields with anatomical information obtained from MRI. In this way spatio-temporal analysis of EEG/MEG can yield useful information about the propagation of inter-ictal spikes in epileptogenic networks. The validation of this approach was systematically assessed in the study of Tanaka et al. (2010) who compared the results of the spatio-temporal source analysis of EEG and MEG of inter-ictal spikes with data obtained by way of iEEG recordings made extraoperatively in 10 patients. This study showed that the analysis based on MEG spikes yield a very similar propagation pattern as observed in iEEG, better than EEG data. It should be noted, however, that the MEG signals were obtained from 102 sites whereas for the EEG only 70 recording sites were used, what may account, at least partially, for the difference in performance between the two sets of recordings.

**Figure 3:**
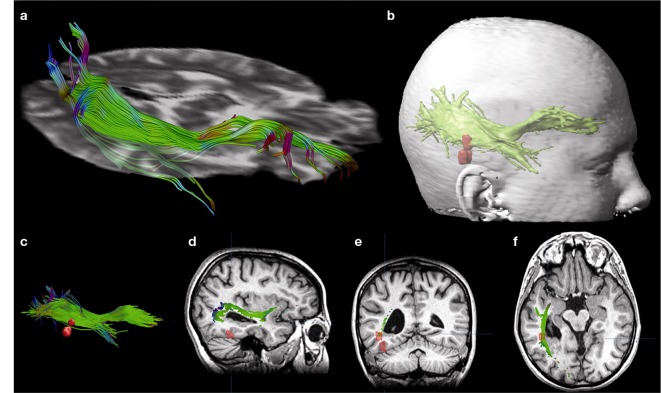

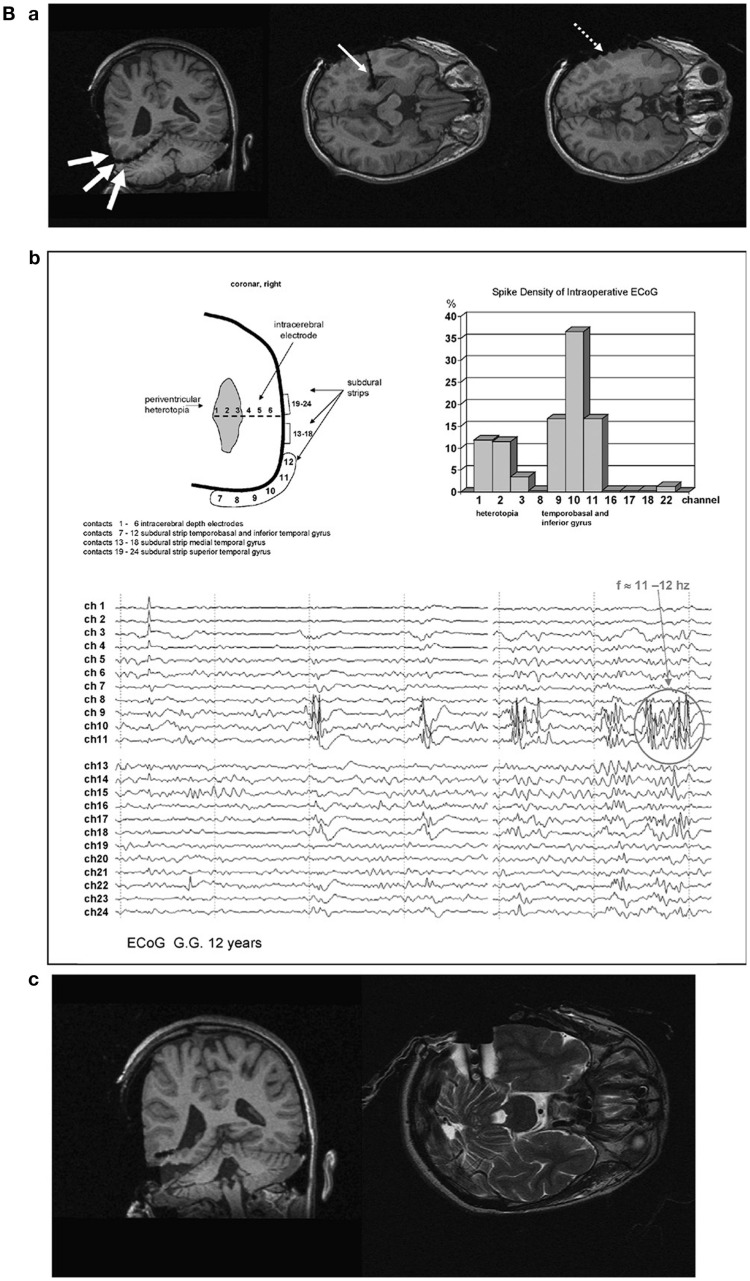
**(A)** (a) Streamtube visualization of the right optic radiation based on diffusion tensor imaging. (b) For navigation, a three-dimensional object representing the optic radiation (wrapping the individual fibers) and two distinct MSI foci (red) are generated. (c) Relation of optic radiation (visualized as streamlines) to MSI foci. (d–f) Sagittal/coronal/axial view of T1-weighted images with registered DTI and MSI data. Localization of focal epileptic activity is below the optic Tract (Stefan et al., 2007). **(B)** (a) Intraoperative electroencephalographic recordings with platinum-electrodes electrode position confirmed by intraoperative T1- and T2-weighted high-field-MR imaging. (b) MSI guided electrode implantation of intra-cerebral depth and subdural electrodes; spike activity in lateral cortex and periventricular heterotopia, the corresponding spike density distribution is shown (upper right). The neocortex shows predominant spike wave activity and 11–12 s^−1^ polyspikes during intraoperative ECoG. (c) Intraoperative MR-imaging after cortical resection of MSI-focus No. II (with platinum-electrodes still *in situ*).

**Figure 4:**
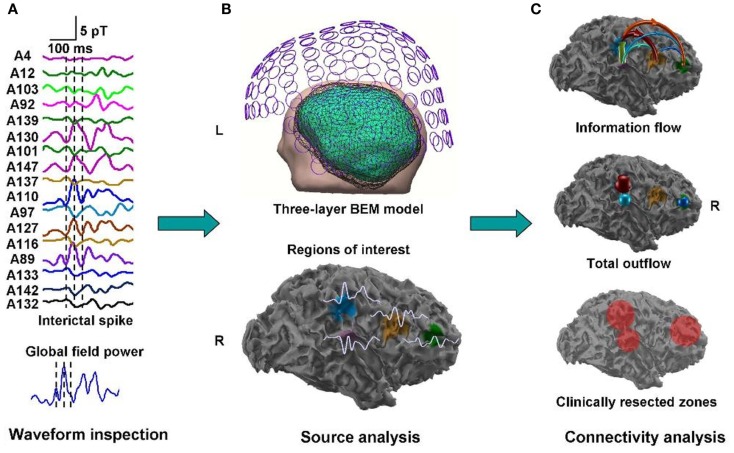
**Estimated direction of propagation of epileptiform activity along neuronal networks (Dai et al., 2012)**.

A clinical example where the value of inter-ictal MEG spikes is illustrated is the case study of a patient with PNH where Magnetic Source Imaging (MSI), i.e., a combination of MEG and co-registered MRI, was applied, and used to guide intraoperative electrocorticography. Thereafter intra-cerebral depth electrodes and subdural strips were implanted guided by the MSI data, which revealed two separated zones of spike activity. A cluster analysis of electrographic recordings of spikes showed two clusters based on source localization using equivalent dipole source models and a realistic volume conductor model; the main cluster arose from the temporal neocortex and not from the PNH area. This led to the assumption that the networks of the temporal cortex distant from the lesion might act as primary epileptogenic network. Accordingly a resection of the temporo-occipital neocortical tissue including the main spike focal area was performed, with excellent postoperative seizure control (Figures 3A,B; Stefan et al., 2007). This led to the definition of the epileptogenic network, including the heterotopia and overlying neocortex, what was essential to assure a positive surgical outcome. Another study (Dai et al., 2012) used also inter-ictal Magnetoencephalography (MEG) to identify epileptogenic networks; these authors estimated cortical sources of spikes using realistic head models, computed the directional connectivity between those sources in patients with pharmaco-resistant epilepsies (different kind of pathologies) who were pre-surgically investigated using intra-cranial electrodes under the guidance of the MEG data, and whose epileptogenic zone was later removed. They found a good overlap between the primary sources of epileptiform spikes and the epileptogenic zones that were later surgically resected, in line with the results obtained by Tang et al. (2003), Shiraish et al. (2005), Stufflebeam et al. (2009), and Wang et al. (2012). In the context of the present discussion on the importance of network analysis in epilepsy, the study of Dai et al. (2012) goes further than previous investigations because these authors applied the DTF, introduced above, using an open-source software package (eConnectome). In this way they estimated the direction of the propagation of the epileptiform activity along the interconnected neuronal networks as illustrated in Figure 4.

### Networks in generalized epilepsies

In the light of the concepts of epileptic neuronal networks and recent experimental findings the dichotomic classification in focal and generalized epilepsy has to be re-evaluated. Indeed it is necessary to reassess the role of epileptic networks in the so-called generalized epilepsies.

#### Are generalized epilepsies actually fast spreading focal epilepsies?

The concept of “primarily generalized epilepsy,” as for example in Childhood Absence Epilepsy (CAE), implies that all brain regions simultaneously would generate spike-and-wave discharges (SWDs) and the associated seizure, classically considered to be triggered by some central process associated with the diffuse cortico-thalamic system according to the “centrencephalic” concept of Jasper and Penfield (1954), or by the interplay between thalamus and cortex as in the corticoreticular hypothesis of Gloor (1968). There is growing evidence, however, that this is a too simplified view, and that in these cases there is a cortical frontal neuronal network where the onset of the SWDs is located. Thus the question may be formulated in a simple way: are SWDs manifestations of a generalized or of a focal process? In the same vein a critical analysis of the classification of epilepsies recommend “abandoning these terms as overall classification categories into which all epilepsies must fit” (Berg and Scheffer, 2011).

**Figure 5:**
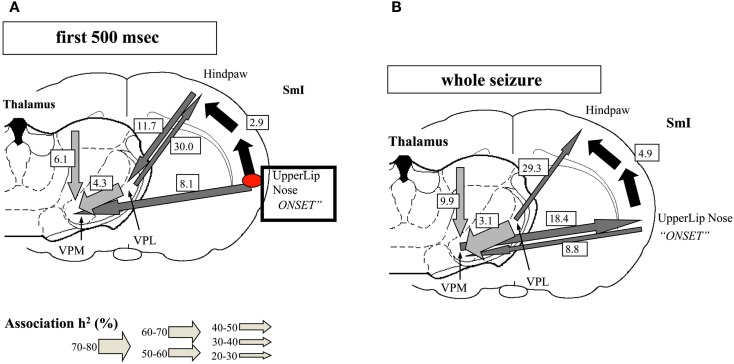
**Evolution of absence seizures in the rat genetic model: (A) corticocortical (represented by the black arrows), intra-thalamic (light gray arrows), and cortico-thalamic (dark gray arrows) interdependencies during spontaneous absence seizures in the WAG/Rij rat as established by the non-linear association *h*^2^ analysis**. The thickness of the arrow represents the average strength of the association, and the direction of the arrowhead points to the direction of the lagging site. The values represent the corresponding average time delays in milliseconds. This example represents the average of 10 seizures of one rat. The relationships are stable for the first 500 ms of the absence seizure. A consistent cortical onset was found in the upper lip and nose area of the somatosensory cortex (SmI), because this site consistently led the other cortical recording sites. The hindpaw cortical area was found to lag by 2.9 ms on average with respect to this focal site. Within the thalamus, the laterodorsal (LD) nucleus was found to consistently lead other thalamic sites. The ventroposterior medial (VPM) nucleus was found to lag behind the ventroposterior lateral (VPL) nucleus, with an average time delay of 4.3 ms. Concerning cortico-thalamic interrelationships, the cortical focus site consistently led the thalamus (VPM), with an average time delay of 8.1 ms. **(B)** The relationships found when the whole seizure is analyzed as one epoch. The same cortical focus as during the first 500 ms was found consistently. Compared with the first 500 ms, the time delay from the cortical focus with respect to the non-focal cortical sites increased. Furthermore, the strength of association between VPL and VPM also increased. The direction of the cortico-thalamic couplings changed. For the non-focal cortical sites, the thalamus was found to lead during all seizures. For the focal cortical site, the cortex was found to lead during two seizures, whereas the thalamus was found to lead during seven seizures (Meeren et al., 2002).

One important hindrance in solving this controversy is that many studies have used inadequate methodologies. Most of the classic observations of “generalized SWDs” (GSWDs) were based on visual inspection of EEG recordings on paper at relatively high speed, as even reported recently (Koutroumanidis et al., 2012). Although the human condition of CAE may differ in some respect from genetic rodent models of Absence epilepsy [Genetic Absence Epileptic Rats of FStrasbourg and Wag/Rij rats (GAERS) = Wistar Albino Glaxo from Rijswijk] the detailed observation of the evolution of the typical SWDs in the latter, using appropriate techniques, allowed Meeren et al. (2002) to identify the focal cortical onset of SWDs as the peri-oral region of the somatosensory cortex (Figure 5). A crucial point is that these local SWDs propagate very quickly throughout the cortex and to the thalamus at the millisecond scale. This fast propagation can only be accurately determined using appropriate analytical methods, such as non-linear regression analysis, at the very beginning of the burst of SWDs; within a few hundreds of milliseconds the propagation to other cortical areas and to the thalamus feeds back to the initial cortical area, what confounds any possibility of determining later where the SWDs had started.

These observations have two important methodological consequences: (1) any method of analysis needs to be reliably applicable to very short signal epochs in the order of <500 ms: (2) the sampling both in time and space has to be very high, at the millimeter and millisecond scales.

This cortical focal onset of SWDs first identified in the cortex of Wag/Rij rat was subsequently further characterized by means of intracortical and intracellular recordings by Polack et al. (2007, 2009) and Pinault (2003) in GAERS, what is in line with the previous observations that the appearance of SWDs in the electrocorticogram precede the corresponding discharges in the thalamus by Seidenbecher et al. (1998). The hypothesis that absence seizures have a focal cortical origin in WAG/Rij rats is also supported by the abolition of SWDs after pharmacological inhibition of the focal region (Sitnikova and van Luijtelaar, 2004; van Luijtelaar and Sitnikova, 2006) and by a series of studies showing abnormal excitability of the cortical focal region (see also Lüttjohann et al., 2011). Furthermore an integrated fMRI/EEG study in GAERS confirmed also the leading role of the somatosensory cortical focal area in SWDs, where the hemodynamic signals were analyzed using Granger causality and DCM along with local field potentials (David et al., 2008).

#### Can the cortical focal origin of SWDs in rat absence models be extrapolated to human patients?

Early classic observations of Bancaud et al. (1974), Rodin et al. (1994), Niedermeyer et al. (1969), and Niedermeyer (1972) suggested that in CAE there was a focal onset of SWDs in the frontal cortex. In the same line the temporal analysis of ictal absence EEG signals revealed a rapid motor involvement in cranio-caudal direction from the ocular/peri-oral regions to the extremities indicating dynamic propagation in a network involving the frontal lobe and motoric system (Stefan, 1982; Stefan and Carter Snead, 1997).

Notwithstanding these and other observations also pointing out in the direction that SWDs in absences are not primarily generalized, this feature was overlooked in the Epilepsy community probably due to the overwhelming weight that the term “ generalized” carries until now in the classification of epileptic seizures. Technical advances of EEG technology (Holmes, 2008), however, gave a new thrust to the search for possible sources of SWDs in the human cortex in patients with CAE. An important advance in this respect was achieved by Holmes et al. (2004, 2010) who recorded scalp EEG signals with a dense-array, 256-channel system in patients with “primary generalized epilepsy,” absence seizures, and carried out source analysis estimating equivalent dipole distributions smoothed by linear inverse methods (LORETA, Pascual-Marqui et al., 2002). The onset of the slow component of SWDs was located at the frontal cortex and the spike at the frontopolar region of orbital frontal lobe. Furthermore a similar analysis (Holmes et al., 2010) in patients with Juvenile Myoclonic Epilepsy (JME) revealed sources in orbitofrontal/medial frontopolar cortex in all patients examined, and in half of the patients sources in basal-medial temporal lobe sources were found. Thus these authors conclude from these observations that JME is “not generalized in the sense of bilaterally diffuse onset.”

**Figure 6:**
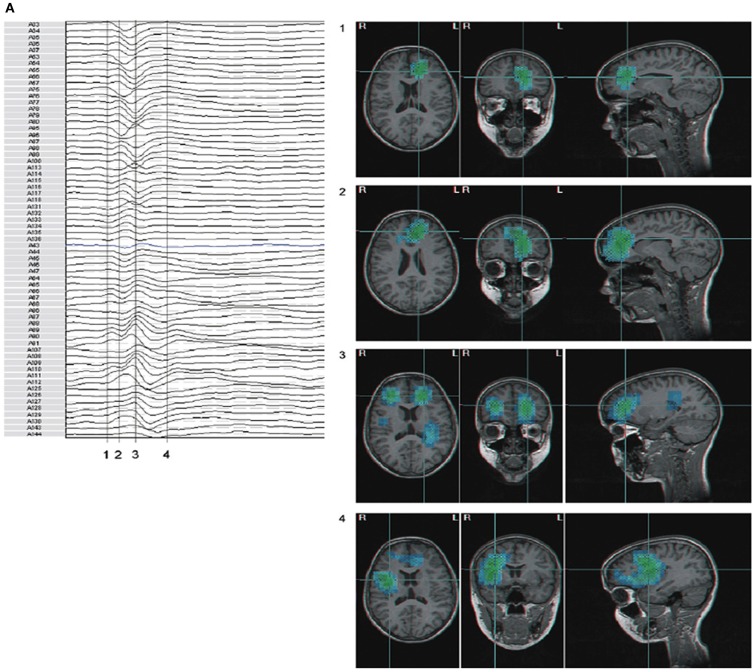

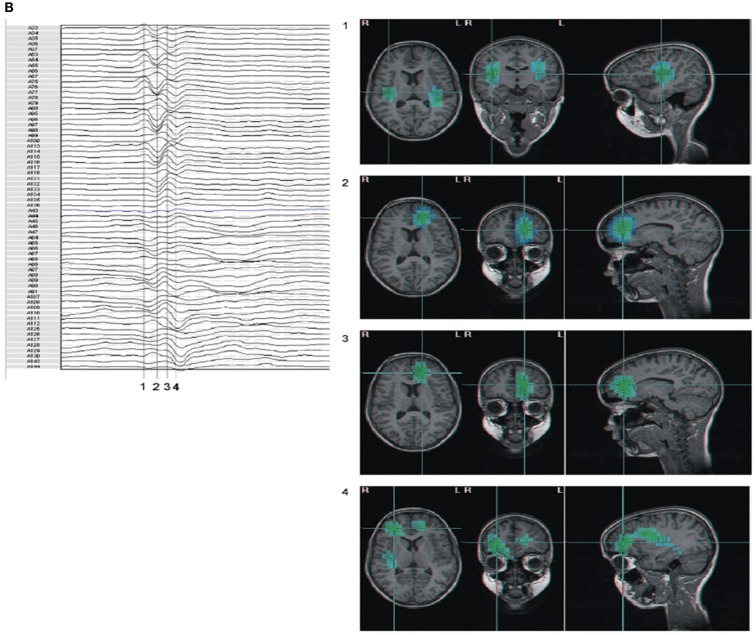
**(A,B)** Beamformer analysis of spike propagation of two MEG spike – wave complexes. Both showed predominant epileptic activity frontal mesial bilateral and perisylvian region (case 5 MAE), however propagation sequence was different (blue to cyan signifies increasing activity). Numbers correspond to time points in the waveform. **(A)** Activity propagated from left frontal mesial area, to larger frontal areas, including polar and basal, bilateral frontal, and subsequently to perisylvian areas; **(B)** in parts reversed propagation sequence compared to **(A)**: bilateral perisylvian origin, propagation to left frontal mesial, and subsequent involvement of bilateral frontal areas. (For interpretation of the references to color in this figure legend, the reader is referred to the web version of the article; Stefan et al., 2009).

Further clinical studies in patients with CAE, JME, and epilepsy with generalized tonic-clonic seizures (GTC) by Stefan et al. (2009) using MEG/EEG, demonstrated regional activations of the fronto polar medial cortex and rapid involvement of other brain areas (Figures 6A,B). The distribution of SWDs in absence patients appeared to involve a prefrontal-insular-thalamic network, whereas in patients with myoclonic components the dominant networks were (pre)motorinsular-thalamic.

Using 204-channel MEG recordings in patients with juvenile absence epilepsy (JAE) and applying dynamic statistical parameter mapping (sSPM) Sakurai et al. (2010) found that the onset of SWDs corresponded to focal cortical activation with secondary activation of posterior cingulate and precuneus, brain structures that belong to the “default mode network (DMN),” at least in some patients.

Since in the great majority of patients with apparently generalized Spike-and-Wave (GSW) and “Idiopathic Generalized Epilepsies” no iEEG recordings are made, several investigators have attempted to determine the role of the thalamus and the cortex in the generation of these discharges using imaging methods, namely fMRI associated with EEG. Using this methodology Salek-Haddadi et al. (2003) studied a patient with IGE and frequent absences and found that generalized SWDs were time-locked with bilateral BOLD activation of the thalamus and cortical deactivation most prominent in the frontal cortex. A similar study by Aghakhani et al. (2004), in a group of patients with IGE and GSWDs described bilateral activation in thalamic regions associated with SWDs, while in the cortex deactivations were observed. Moeller et al. (2009) investigated not only GSWDs in patients with absence seizures but also triggered photoparoxysmal responses (PPRs) using EEG based coherent source dynamic imaging and BOLD signals. Partial directed coherence analysis indicated that the thalamus appeared to act as “pacemaker” of GSWDs in absence seizures; in contrast PPRs could be accounted for by an activation of the occipital cortex that propagates along cortical networks to frontal areas.

Carney et al. (2012) used also EEG-fMRI to further investigate the role of the frontal cortex in absence seizures. They identified two major patterns of frontal cortical BOLD signal change following onset of SWDs using event-related time course analyses, in the dorsolateral prefrontal cortex (DLPF): one group showed a pronounced and prolonged positive cortical BOLD signal change, whereas another group displayed a less pronounced BOLD increase followed by a predominant negative BOLD signal change. Similar patterns were found in the midline and lateral parietal cortex, caudate, and thalamus. They report also evidence of BOLD signal changes that precede the Spike-and-Wave onset, particularly in the mesial frontal cortex, parietal cortex, and precuneus, but not in the thalamus.

These authors suggest that the differences in frontal cortical BOLD associations with onset of absences may have phenotypic implications. This implies that group-averaged data have to be interpreted with caution, and that individual recordings have always to be examined.

A general comment about the findings of these studies in the light of the experimental evidence obtained in animal models of absences, is that the dynamics of the onset and early propagation of SWDs from the cortical onset zone to the thalamus takes place at a very fast pace. Only during a couple of hundreds of milliseconds there is a sustained flow of SWD signals from cortex to thalamus; after that the cortico-thalamo-cortical loop is entrained in the oscillations and it is not possible anymore to identify where the onset was situated. Therefore BOLD signals, that have much longer time constants don’t have the adequate time resolution to catch the dynamical onset event of SWDs. The changes in BOLD signals put in evidence by all the studies referred to above, however, are consistent: during a burst of SWDs there is activation of thalamus and deactivation of cortical areas, what represents the steady-state condition of the underlying neuronal networks during a SWD burst, but are not informative regarding the identification of the onset dynamics of SWDs.

#### Does network analysis reveal abnormalities of the inter-ictal state in patients with IGE?

Children with CAE can display besides SWDs associated with absences also SWDs that may be clinically silent. Li et al. (2009) using EEG-fMRI analyzed both types of GSWDs, inter-ictal and ictal and reported that both types were associated with changes of BOLD signal in the basal ganglia-thalamo-cortical loop, but whereas the ictal type showed widespread and symmetrical deactivation in the cortex, the inter-ictal type showed predominant cortical activation. The authors advance the hypothesis that the cortical deactivation would be the substrate for the abrupt loss of consciousness of the absences. Interestingly Luo et al. (2011) investigated, using fMRI, the inter-ictal state also in patients with absences but taking care of avoiding that SWDs were present during the recording, i.e., in the resting state. Cross-correlation functional connectivity analysis revealed decreased integration within the DMN in the absence epilepsy patients, in particular a decrease of functional connectivity among the frontal, parietal, and temporal lobe. It is to be investigated whether this DMN abnormalities may be related to cognitive impairments in these patients.

### New approaches to delineate epileptogenic networks also with regard to guiding therapy

In the previous sections we considered how various modern methodologies can be applied to improve the delineation of the epileptogenic zone having in mind that the latter should be envisaged as an *epileptogenic neuronal network*, and we discussed several typical clinical cases. In the modern conceptualization of epilepsy one has to evolve from “zones to networks” paraphrasing Laufs (2012). The identification of epileptogenic networks is, of course, of paramount importance in order to improve planning of resective surgery, and also to guide targeted therapies with the aim of controlling abnormal activity in relevant hubs and nodes within the epileptogenic network.

In the context of surgical planning in patients with medically intractable epilepsies one crucial aspect is to optimize the placement of depth electrodes (iEEG), such that the brain space of interest may be appropriately explored. Currently the possibilities offered in this respect by combining EEG and fMRI are the object of active research (Vulliemoz et al., 2010). A topical issue is whether fMRI may have a relevant added-value to classic EEG scalp recordings regarding the analysis of epileptogenic networks. Of course fMRI being non-invasive would represent an important practical tool in this context. Also in association with iEEG, fMRI might be valuable since it permits to extend the scope of the search for biomarkers of epileptic activity to the whole brain, compensating the spatial sampling limitations of iEEG. It is important to emphasize that EEG-fMRI studies should take into account not only the question of localization within brain space of “hot spots” of epileptiform activities but also the dynamic features of these activities, i.e., the flows of propagation and the corresponding time delays. In many investigations these dynamic aspects are still too little explored.

Some promising findings, however, have been reported. In a study of patients with focal epilepsies Jacobs et al. (2009) demonstrated the occurrence of BOLD changes associated with inter-ictal spikes recorded at the scalp that preceded the latter by a few seconds. This early BOLD response may be interpreted as resulting from changes in neuronal activity in epileptogenic neuronal networks situated deep in the brain that are not reflected at the level of the scalp EEG.

The study of Fahoum et al. (2012) addressed the same question by investigating the distribution of cortical and subcortical hemodynamic changes associated with inter-ictal spikes (IEDs) recorded in scalp EEG in patients with different epileptic conditions (temporal lobe-TLE, frontal lobe-FLE, and posterior quadrant-PQE epilepsies) using a similar EEG-fMRI approach. These authors modeled the BOLD response to the IEDs using the timing of the epileptiform events as regressor convolved with a series of Hemodynamic Response Functions (HRFs) consisting of gamma functions peaking at successive delays. Without going here into details the main findings of the analyses showed widespread clusters of activation and deactivation in TLE and FLE patients, while in PQE only deactivations clusters were found, that reached brain areas outside the presumed epileptogenic zone. The largest activations both in TLE and FLE patients were found bilaterally in mid-cingulate gyri. All patient groups showed deactivations of DMN regions, particularly in TLE patients (inferior parietal lobules, posterior cingulate cortex, and precuneus bilaterally). The involvement of the mid-cingulate gyri likely reflects the rapid propagation of epileptic activity from sources in temporal and frontal areas. The pathophysiological significance of this finding is, however, not yet clear. Nonetheless this may be a pointer to further investigate whether these networks involving the cingulated cortex may be interesting targets for therapeutic interventions.

Simultaneous intra-cranial recordings with an appropriate spatial sampling, however, are necessary to clarify these functional relationships revealed by the scalp IEDs – fMRI analyses. In a study of patients with FCDs Thornton et al. (2011) made a comparison between EEG-fMRI signals associated with inter-ictal epileptiform discharges (IEDs) in iEEG recordings in order to delineate in a classic way the seizure onset zone (SOZ). These authors studied also the surgical outcome of these patients. About 5 of 11 patients showed IED-related BOLD signals that were concordant with the electrophysiologically determined SOZs, that in these patients had a limited extent. Six of 11, however, did not display this concordance and the IED-related BOLD signals revealed more widespread epileptogenic regions in comparison with the extent of the SOZ delineated based only on iEEG data. Most interesting the five former patients had a good surgical outcome, but this was not the case for the latter 6. These findings suggest that EEG-fMRI may be useful to identify patients with extensive epileptogenic networks that extend beyond those delineated using only classic iEEG. This information may contribute to making decisions concerning surgical resections more appropriately.

Thus EEG/fMRI studies may be helpful in order to plan more efficiently iEEG recordings and to estimate more accurately the extent of epileptogenic neuronal networks. One critical note should be added: many EEG-fMRI studies tend to focus mainly on localizations rather than on dynamics. In general these studies don’t take into account time-delays between different hubs and nodes within extensive epileptogenic networks. In order to obtain this information one has to resort to electrophysiological measurements, given the low time resolution of fMRI signals. To obtain a comprehensive picture of epileptogenic networks it is essential to uncover the dynamics of the propagation of epileptic activity in these networks.

It is interesting to note that these techniques are being applied also to find whether the pattern of functional connectivity of neuronal networks may yield relevant information about epileptogenic networks during inter-ictal resting states. Using non-linear correlation analysis Bettus et al. (2011) showed in TLE patients a general increase of iEEG signal interdependencies (specific for the beta frequency band) in regions affected by electrical epileptiform abnormalities relative to non-affected areas, whereas the opposite pattern was found for functional connectivity measured using fMRI signals. This latter finding may be due to anomalies in metabolism and in neurovascular coupling (blood-brain-barrier permeability) in epileptogenic networks that may be affected in TLE. Such anomalies have been also shown in animal models of TLE and in human patients by means of MR diffusion imaging – tractography (Yogarajah et al., 2008). Bettus et al. (2011) suggest that this increase in beta frequency interdependencies in epileptogenic networks “could be a reliable pathological marker of epileptic processes.” This needs further confirmation.

With respect to locally targeted therapies experimental work in animal models reveals some interesting novel perspectives for future therapeutic interventions, for example with the purpose of delivering anticonvulsants locally into specific hubs of epileptogenic networks as proposed by Löscher and collaborators (Bröer et al., 2012). Strategies for neuromodulation aiming at the therapeutic control of epileptogenic networks are being considered besides local drug delivery, such as local electrical stimulation, transcranial magnetic stimulation, stem cells transplantation, and gene therapy (see review Al-Otaibi et al., 2011). Regarding deep electrical brain stimulation (DBS) Kahane and Depaulis (2010) stress the importance of gaining a better understanding of the functional properties of epileptogenic neuronal networks in which seizures originate and propagate, as much as of the mechanisms by which neurostimulation works, in order to define the types of DBS that may be effective. The need of acquiring a comprehensive insight in these functional epileptogenic networks applies to all strategies to develop novel targeted therapies.

## Conclusion

The interest on neuronal network analysis in epilepsy has gained strength with the use of high resolution recording techniques and signal analytical methodologies that opened up the possibility of studying dynamic brain states with high resolution both in space and time. In addition to invasive EEG recordings, non-invasive recording techniques like MEG/EEG and EEG-fMRI are being used more and more to identify network involvements in various epileptic conditions and age dependent syndromes, both structurally and dynamically. In some cases diverse techniques are used in a complementary way. A typical example is the study of Vaudano et al. (2012) who made ictal MEG and EEG-fMRI recordings in a patient with reading epilepsy. Using this information combined with DCM, they found evidence for a causal link between activity in the left piriform cortex and the seizures elicited by reading. More research is needed to integrate this kind of findings with the detection of relevant biomarkers of epileptogenic zones, that have been recently described such as (fast)ripples (HFOs, Jacobs et al., 2012; Jefferys et al., 2012) in order to refine systematic network analyses in all sorts of epileptic patients.

In the last decade the development of new methodologies to analyze the dynamics of neuronal networks has gained momentum, and has yield a wide range of computer tools that are being tested in clinical and experimental environments. Using such tools, many of which were discussed above, new insights are emerging.

One of these is that the concept that some types of epilepsy are “generalized” is outdated. Even in cases of IGE and CAE there is now compelling evidence that the seizures start in a well defined brain area and spread at great speed to connected brain areas recruiting specific neuronal networks into typical oscillatory behavior. This conclusion is now supported by high resolution human EEG and MEG data, by animal experimental electrophysiological and fMRI data. Thus the dividing line between “generalized” and “focal” epilepsies recedes on the face of new relevant neuronal network analyses. In the classification of seizures, it is important, in addition to the localization of seizure onset, to identify cortical and subcortical network involvement and the speed of propagation. This implies that a neurophysiological seizure classification approach should include the identification of the predominant involved networks, within one hemisphere or involving both. This conclusion is in line with Laufs’ (2012) conclusion that the concept that epilepsies should be considered as resulting from disturbed network interactions that implies “multitargeted treatments.” A major challenge will be to perform connectivity analysis in order to identify the primary epileptogenic source, or “hub,” in complex multifocal epilepsies in order to optimize tailoring surgical resections and/or targeted therapies in epileptic patients.

## Conflict of Interest Statement

The authors declare that the research was conducted in the absence of any commercial or financial relationships that could be construed as a potential conflict of interest.
